# Development of Breast Cancer Spheroids to Evaluate Cytotoxic Response to an Anticancer Peptide

**DOI:** 10.3390/pharmaceutics13111863

**Published:** 2021-11-04

**Authors:** Marco Cavaco, Patrícia Fraga, Javier Valle, David Andreu, Miguel A. R. B. Castanho, Vera Neves

**Affiliations:** 1 Department of Biochemistry, Instituto de Medicina Molecular João Lobo Antunes, Faculdade de Medicina, Universidade de Lisboa, Av. Prof. Egas Moniz, 1649-028 Lisbon, Portugal; mcavaco@medicina.ulisboa.pt (M.C.); pfraga@medicina.ulisboa.pt (P.F.); macastanho@medicina.ulisboa.pt (M.A.R.B.C.); 2 Proteomics and Protein Chemistry Unit, Department of Experimental and Health Sciences, Pompeu Fabra University, Dr. Aiguader 88, Barcelona Biomedical Research Park, 08003 Barcelona, Spain; javier.valle@upf.edu (J.V.); david.andreu@upf.edu (D.A.)

**Keywords:** 3D cell culture, anticancer peptides, breast cancer, cell monolayers, preclinical studies, spheroids

## Abstract

Breast cancer (BC) is the most commonly diagnosed cancer in women and one of the most common causes of cancer-related deaths. Despite intense research efforts, BC treatment still remains challenging. Improved drug development strategies are needed for impactful benefit to patients. Current preclinical studies rely mostly on cell-based screenings, using two-dimensional (2D) cell monolayers that do not mimic in vivo tumors properly. Herein, we explored the development and characterization of three-dimensional (3D) models, named spheroids, of the most aggressive BC subtypes (triple-negative breast cancer-TNBC; and human-epidermal growth receptor-2-HER2+), using the liquid overlay technique with several selected cell lines. In these cell line-derived spheroids, we studied cell density, proliferation, ultrastructure, apoptosis, reactive oxygen species (ROS) production, and cell permeabilization (live/dead). The results showed a formation of compact and homogeneous spheroids on day 7 after seeding 2000 cells/well for MDA-MB-231 and 5000 cells/well for BT-20 and BT-474. Next, we compared the efficacy of a model anticancer peptide (ACP) in cell monolayers and spheroids. Overall, the results demonstrated spheroids to be less sensitive to treatment than cell monolayers, revealing the need for more robust models in drug development.

## 1. Introduction

Over the last decade, breast cancer (BC) diagnosis and treatment have significantly improved, resulting in better disease management. Nevertheless, BC is still one of the leading causes of cancer-related deaths among women worldwide [1]. 

The classification of BCs into different subtypes is important to select adequate therapeutic options and evaluate prognosis, with the histological profile as one of the most important criteria. BCs can be classified into invasive ductal carcinoma (80–85% of patients), invasive lobular carcinoma (10–15%), and ductal/lobular carcinoma (5–10%) [2,3]. The occurrence of two molecular targets, namely estrogen-receptor α (ERα) and epidermal growth factor receptor-2 (HER2), constitutes another classification criterion [4]. Erα is expressed in 75% of invasive BCs, and it is closely related to the expression of the progesterone-receptor (PR) [5,6]. HER2 is amplified or overexpressed in 15–20% of BCs [7,8]. Finally, triple-negative breast cancer (TNBC), which corresponds to approximately 10–15% of BCs, is characterized by the lack of ER/PR and HER2 expression [9,10].

In the last decade, significant advances have been made in the understanding of cancer onset and survival, and in the development of new therapeutic platforms allowing the development of new therapeutics against these more aggressive BC subtypes, namely HER2+ and TNBC [4,6]. However, only a small percentage of drugs have advanced into the clinic and are currently in use [11]. In the early stages, both BC subtypes are manageable; however, in advanced stages, therapy is based on palliative care, which underscores the lack of effective drugs [12]. 

The development of new drugs is a demanding and time-consuming process [13]. Usually, it encompasses several in vitro and in vivo screens before assessment in humans. To date, the evaluation of a new drug in an in vitro setting relies primarily on cell-based assays, which provide an easy-to-use, fast, and cost-effective tool [14]. Most of these assays use traditional two-dimensional (2D) cell monolayers, cultured on flat and rigid substrates [14]. Although useful, these cultures do not adequately reproduce the natural three-dimensional (3D) cell microenvironment [15,16,17]. In cancer research, the tumor microenvironment is particularly important, given unique features such as the existence of hypoxic areas, production of extracellular matrix, intercellular interactions, and growth factor exchange [18]. Consequently, the lack of similarities between 2D cell culture models and the in vivo setting might be one of the main reasons for the high percentage of drugs failing clinical trials, albeit promising in early development stages [19,20,21]. 

In contrast to 2D cell models, it has been suggested that 3D models are more representative of the actual in vivo tumor microenvironment [22,23,24,25,26,27], which makes them promising tools for drug development. Numerous 3D culture methods have been studied to generate these models based on (1) the application of automated forces (e.g., centrifugation, spinning, and rotation), (2) hydrogels, and (3) gravity (e.g., hanging drop culture, and liquid overlay culture) [28,29,30]. Based on these different techniques, researchers have been developing spheroids using different cancer cell types and matrices to accurately study chemotherapeutic drugs [28,31,32,33,34,35,36,37,38,39,40,41]. 

This work focuses on the development of BC spheroids for TNBC (MDA-MB-231 and BT-20, which lack common target receptors and differ in proliferation and metastization capability) and HER2+ (BT-474, which expresses growth receptors and presents a high proliferative rate, as well as a relatively high rate of cell loss) cell subtypes highly applied in preclinical studies with chemotherapeutic agents [42]. The liquid overlay culture technique, which allows the formation of pseudo-microtissues, also called spheroids, is based mostly on cell seeding (gravity) in an untreated round-bottomed well, and was chosen as a simple and fast procedure capable of generating highly homogeneous and reproducible spheroids. During protocol optimization, each cell line-derived spheroid was thoroughly characterized by evaluation of cell density, metabolic activity, cell permeabilization (live/dead), apoptosis, oxidative stress, proliferation, and ultrastructure, providing a privileged vantage point over other spheroid production protocols. Such well-characterized BC spheroids provide a realistic setting of the tumor biochemical and biophysical microenvironment vis-à-vis drug testing.

After selecting optimal conditions to develop stable and homogenous spheroids, we compared the efficacy of an anticancer peptide (ACP), vCPP2319 [43,44], in cell monolayers and spheroids. Therapeutic peptides such as vCPP2319 have been explored as a potential alternative to classical chemotherapeutic agents for advantages, such as efficiency, specificity and affinity, minimal drug-drug interactions, and biological and chemical diversity [45,46,47], and like chemotherapeutic agents, lack proper characterization in more robust models, such as spheroids. In this study, vCPP2319 efficacy was compared with PepH3, known for penetration of cellular barriers but lacking anticancer activity [43]. The proposed comparison will demonstrate the importance of using complex cell culture models for an accurate evaluation of drug efficacy. 

## 2. Materials and Methods

### 2.1. Chemicals and Materials

3,6-dioxa-1,8-octanedithiol (DODT) was purchased from Sigma-Aldrich (Spain). Solvents and reagents for peptide synthesis (*N,N*-dimethylformamide (DMF), *N,N*-diisopropylethylamine (DIEA), dichloromethane (DCM), trifluoroacetic acid (TFA), *N,N*-diisopropylcarbodiimide (DIPCI), and triisopropylsilane (TIS)), as well as HPLC-grade acetonitrile (CAN) were purchased from Carlo Erba-SDS (Sabadell, Spain). Fmoc-protected amino acids, Fmoc-Rink amide (MBHA) resin, *N*-hydroxybenzotriazole (HOBt), and 2-(1H-benzotriazol-1-yl)-1,1,3,3-tetramethyluronium hexafluorophosphate (HBTU) were purchased from Iris Biotech (Marktredwitz, Germany). 

Dulbecco’s modified Eagle medium (DMEM), trypsin-EDTA, fetal bovine serum (FBS), l-glutamine, and penicillin-streptomycin antibiotic solution (pen/strep solution) were purchased from Gibco/Thermo Fischer (Waltham, MA, USA). Eagle’s minimum essential medium (EMEM) was purchased from Sigma-Aldrich (Madrid, Spain). CellTiter-Blue^®^ cell viability reagent was purchased from Promega (Madrid, Spain). CellEvent^TM^ ReadyProbe^®^, CellRox^®^ Deep Red reagent, and Live/Dead^TM^ viability/cytotoxicity kit were purchased from Invitrogen/Thermo Fisher (Waltham, MA, USA).

### 2.2. Cell Culture

TNBC cell lines MDA-MB-231 (ATCC^©^ HTB-26^TM^) and BT-20 (ATCC^©^ HTB-19^TM^) were cultured as a monolayer in DMEM and EMEM, respectively. The HER2+ cell line BT-474 (ATCC^©^ HTB-20^TM^) was cultured as a monolayer in EMEM. Additionally, we added 10% FBS and 1% pen/strep solution to the respective medium, following the instructions from the manufacturer. All cells grew in a humidified atmosphere at 37 °C and 5% CO_2_ (MCO-18AIC (UV), Sanyo, Osaka, Japan). The medium was changed every other day.

### 2.3. Tumor Spheroid Generation

Cells were allowed to grow until 80% confluence in a 75 cm^2^ T-flask. Then, spheroids were generated using the liquid overlay technique. Briefly, cells were seeded in an ultra-low attachment 96-well round-bottomed plate with covalently bonded hydrogel that minimizes cell attachment (Ref. 7007, Corning, NY, USA). For spheroid optimization, a seeding density of 50 to 10,000 cells/well was used, after which the optimal regimen conditions were established for each spheroid and used for all remaining assays: 2000 cells/well for MDA-MB-231 and 5000 cells/well for BT-20 and BT-474. A cold (4 °C) solution of GelTrex^®^ LDEV-free (Alfagene/Thermo Fisher, Waltham, MA, USA) diluted in the respective medium was added into each well (2%, *v*/*v*) after 24 h. Then, microplates were agitated (185 rpm for 20 min) and put in the incubator under culturing conditions until the day of the experiment. During the optimization assay, spheroids were cultured for 14 days without changing or adding medium. After determination of the best regimen conditions, a 7-day incubation period was considered optimal and used in subsequent studies. 

### 2.4. Characterization of Spheroid Diameter and Morphology

During spheroid optimization, diameter and morphology were monitored daily from day 1 (i.e., the day after addition of cellular matrix) to day 14, using the wide field fluorescence microscope Zeiss Axiovert 200M (Carl Zeiss, Oberkochen, Germany) equipped with an EC Plan-Neofluar ×10 dry objective (0.30 numerical aperture) and a Leica DFC450 camera. The diameter of each spheroid was measured with the “*cellular analysis*” algorithm using Fiji software. At least three replicates on different days were used.

### 2.5. Metabolic Activity Assessment

The metabolic activity of spheroids was monitored daily from day 1 to 14 during the optimization procedure, using CellTiter-Blue^®^ cell viability assay, according to the manufacturer’s instructions. Briefly, on the day of the experiment, 20 µL of CellTiter-Blue^®^ reagent was added to each well and incubated for 6 h in culturing conditions. The fluorescence intensity was measured at 560 nm excitation and 590 nm emission using the Varioskan^TM^ LUX microplate reader (Thermo Fisher, Waltham, MA, USA). At least three replicates on different days were used.

### 2.6. Apoptosis Determination

Activation of apoptosis was measured with a CellEvent^TM^ ReadyProbe^TM^, according to the manufacturer’s instructions. Briefly, on the day of the experiment, spheroids were harvested and transferred into 6-well flat-bottomed plates (Corning, NY, USA), washed twice with 1× PBS (pH 7.4), and incubated with the probe for 60 min. Then, we transferred the spheroids to 8-well µ-slides (Ibidi, Gräfelfing, Germany) and used a confocal point-scanning Zeiss LSM 880 microscope (Carl Zeiss, Oberkochen, Germany) equipped with an alpha Plan-Apochromat ×20 dry objective (0.80 numerical aperture) to image them. We selected a 488-nm Ar laser to excite the probe. The images were recorded in the normal confocal mode at 3440 × 3440 resolution using ×0.6 zoom. To acquire and process all images, we used Zen and Fiji software. At least three replicates on different days were used. 

### 2.7. Oxidative Stress Measurement

The production of reactive oxygen species (ROS) was measured using a CellRox^®^ Deep Red reagent, according to the manufacturer’s instructions. Briefly, on the day of the experiment, spheroids were harvested and transferred into 6-well flat-bottomed plates (Corning, NY, USA), washed twice with 1× PBS (pH 7.4), and incubated with CellRox^®^ reagent (5.0 µM) for 60 min. Then, we transferred the spheroids to 8-well µ-slides (Ibidi, Gräfelfing, Germany) and used a confocal point-scanning Zeiss LSM 880 microscope (Carl Zeiss, Oberkochen, Germany) equipped with an alpha Plan-Apochromat ×20 dry objective (0.80 numerical aperture) to image them. We selected a 633-nm HeNe633 laser to excite the probe. The images were recorded in the normal confocal mode at 3440 × 3440 resolution using ×0.6 zoom. To acquire and process all images, we used Zen and Fiji software. At least three replicates on different days were used.

### 2.8. Spheroid Viability Monitoring

Spheroid viability was monitored using a Live/Dead^TM^ viability/cytotoxicity kit, according to the manufacturer’s instructions. Briefly, on the day of the experiment, spheroids were harvested and transferred into 6-well flat-bottomed plates (Corning, NY, USA), washed twice with 1× PBS (pH 7.4), and incubated with ethidium homodimer-1 (EthD-1, 4.0 µM in 1× PBS (pH 7.4)) and calcein-AM (2.0 µM in 1× PBS (pH 7.4)) for 45 min. Then, we transferred the spheroids to 8-well µ-slides (Ibidi, Gräfelfing, Germany) and used a confocal point-scanning Zeiss LSM 880 microscope (Carl Zeiss, Oberkochen, Germany) equipped with an alpha Plan-Apochromat ×20 dry objective (0.80 numerical aperture) to image them. We selected a 488- and 514-nm Ar laser to excite the probes. The images were recorded in the normal confocal mode at 3440 × 3440 resolution using ×0.6 zoom. To acquire and process all images, we used Zen and Fiji software. At least three replicates on different days were used.

### 2.9. Transmission Electron Microscopy

Cell organization and ultrastructure within spheroids were observed by transmission electron microscopy (TEM). Established spheroids were washed in 0.1 M Na cacodylate buffer (pH 7.4) and fixed in 2.5% glutaraldehyde for 24 h at 4 °C. Specimens were then washed 3× (10 min), post fixed for 1 h with 1% osmium tetroxide, and washed 3×, all steps in Na cacodylate buffer. Then, spheroids were dehydrated in ethanol (70%, 95%, 100%) and propylene oxide and infiltrated with propylene oxide and EPON resin mixture (2:1, 1:1, 1:2 for 1 h). Subsequently, they were embedded in EPON resin overnight at room temperature and incubated for 2 days at 60 °C. Next, we cut the specimens using a UC7 ultramicrotome (Leica) into thin sections (70 nm) and stained with uranyl acetate and lead citrate. The cells’ ultrastructural organization within the spheroids was observed using TEM (Hitachi H-7000 microscope) at 100 kV acceleration voltage. Micrographs were made using a MegaView III camera placed in a side position.

### 2.10. Peptide Synthesis and Purification

PepH3 (AGILKRW-amide) and vCPP2319 (WRRRYRRWRRRRRQRRRPRR-amide) were both produced by solid phase peptide synthesis on Rink-amide ChemMatrix resin at 0.1 mmol scale in a Gyros Prelude (Tucson, AZ, USA) instrument running Fmoc protocols (Table 1). Trifunctional residue side chain protections were tert-butyloxycarbonyl (Trp), tert-butyl (Glu, Ser, Thr, and Tyr), and NG-2,2,4,6,7-pentamethyldihydrobenzofuran-5-sulfonyl (Arg). Couplings were achieved with an 8-fold molar excess of both Fmoc amino acid and HBTU, plus a 16-fold molar excess of DIEA, in DMF. After chain assembly, treatment with TFA/H_2_O/DODT/TIS (94:2.5:2.5:1 *v*/*v*, 90 min, r.t.) achieved full deprotection and resin cleavage. The crude peptide was precipitated from the TFA solution by cold ether addition and centrifugation (4000× *g*, 4 °C for 20 min), and the pellet was dissolved in H_2_O and lyophilized.

Analysis of peptide purity was performed by RP-HPLC (Luna C18 column, 4.6 × 50 mm, 3.0 µm; Phenomenex, Torrance, CA, USA) using a linear gradient of solvent B (0.036% TFA in MeCN) into A (0.045% TFA in H_2_O) at 1 mL/min flow rate and with 220 nm UV detection. Preparative purification was performed by RP-HPLC (Luna C18 column, 21.2 × 250 mm, 10.0 µm; Phenomenex) using a linear gradient of solvent B (0.1% TFA in MeCN) into A (0.1% TFA in H_2_O) at 25 mL/min flow rate and with 220 nm UV detection. Molecular mass determination was performed by LC-MS (XBridge C18 column, 4.6 × 150 mm, 3.5 µm; Waters, Madrid, Spain) using HCOOH/MeCN (0.08% *v*/*v*) into HCOOH/H_2_O (0.1%, *v*/*v*) over 15 min at 1 mL/min. HPLC-homogeneous fractions with the expected mass were combined and lyophilized. One millimole peptide stocks in H_2_O were stored at −20 °C.

### 2.11. Cell Viability Measurement on Cell Monolayers

Peptide cytotoxicity towards MDA-MB-231, BT-20, and BT-474 cell monolayers was determined using a protocol described elsewhere [43]. Different peptide concentrations (0.05–100.0 μM range, in medium), commonly applied in viability screenings, were incubated for 24 h in culturing conditions. IC_50_ values were obtained using GraphPad Prism 7.0 software (GraphPad Software, San Diego, CA, USA) and the log(inhibitor) vs. normalized response. At least three replicates on different days were performed.

### 2.12. Cell Viability Measurements on Spheroids

For MDA-MB-231, and for both BT-20 and BT-474, initial densities of 2000 and 5000 cells/well were used, respectively, with 7-day incubation in all cases. After spheroid formation, cells were treated with increasing peptide concentrations (0.05–100.0 µM, in medium) up to 5 days in culturing conditions without medium change, and cell viability was assessed using the CellTiter-Blue^®^ assay, as described above. A 5-day incubation was used to assess the period necessary for best anticancer activity. IC_50_ values were obtained using GraphPad Prism 7.0 software (GraphPad Software, San Diego, CA, USA) and the log(inhibitor) vs. normalized response. At least three replicates on different days were performed.

### 2.13. Evaluation of Peptide Activity on Spheroids 

The activity was evaluated using the parameters described above for spheroid optimization: apoptosis induction, oxidative stress, and cell permeabilization (live/dead). Briefly, spheroids were incubated for 5 days in culturing conditions with peptide at the IC_50_ value corresponding to the cell line, without medium change, and the specific assay was performed afterward. To compare fluorescence intensity among different spheroids, corrected total cell fluorescence (CTCF) was calculated using Equation (1):

(1) CTCF=Integrated Density−(Area of selected spheroid×Mean Fluorescence of background readings)

At least three replicates on different days were used. 

### 2.14. Statistical Analysis

Results were expressed as means ± standard deviation (SD) of *n* independent experiments. All experiments were performed at least in triplicate on three different days. Then, a one-way ANOVA was applied. Tests were two-sided and the nominal level of significance was * *p* < 0.05, ** *p* < 0.01, *** *p* < 0.001, and **** *p* < 0.0001.

## 3. Results

### 3.1. Optimization of Spheroid Generation

The optimization of MDA-MB-231, BT-20, and BT-474 spheroids was carried out using an initial cell density of 50 to 10,000 cells/well in the presence of 2% (*v*/*v*) GelTrex^®^ over 14 days. Diameter and metabolic activity were evaluated daily. In addition, at days 7 and 14, apoptotic cells, ROS production, and cell permeabilization (live/dead) were monitored. All of these parameters were used to set the best regimen condition for each cell line. 

#### 3.1.1. MDA-MB-231 Cell Line

**Figure 1:**
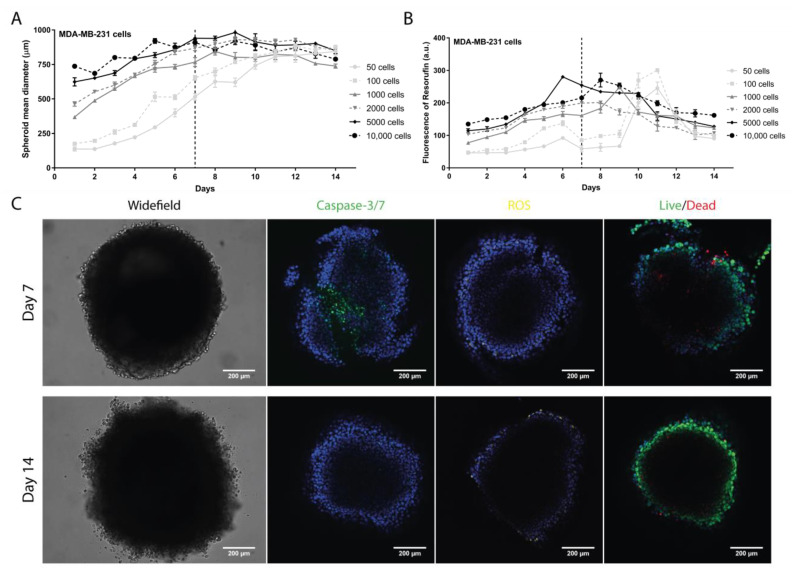
Evaluation of the MDA-MB-231 spheroid formation. (**A**) Spheroid diameter (µm) daily monitoring over 14 days measured with a widefield fluorescence microscope. (**B**) Spheroid cell metabolic activity daily evaluation over 14 days by measuring the fluorescence emission of resorufin with a microplate reader. (**C**) Representative images of parameters evaluated on days 7 and 14 at 2000 cells/well with a confocal point-scanning Zeiss LSM 880 microscope. In the apoptosis determination, apoptotic cells are marked in green; in the oxidative stress measurement, reactive oxygen species (ROS) are marked in yellow; and in the cell viability/mortality evaluation, intact cells (live) are marked in green and permeabilized cells (dead) in red. In all experiments, cell nucleus is marked in blue. Scale bar = 200 µm. Graphs represent at least three biological repeats.

For MDA-MB-231, a progressive increase in diameter was observed until day 7 for all cell densities tested (Figure 1A). On day 14, a slight decrease for 1000, 2000, 5000, and 10,000 cells/well was observed. In contrast, for 50 and 100 cells/well, the diameters continued to increase until day 14. The metabolic activity reached a maximum between day 7 and day 9, followed by a significant decrease until day 14 (Figure 1B). The exceptions were 50 and 100 cells/well, where maximum metabolic activity was registered on day 11 with an abrupt metabolic decrease until day 14.

The best results were observed for 2000 cells/well, for which a gradual increase in diameter and metabolic activity were obtained until day 7, followed by a stabilization of diameter and increase in metabolic activity between days 7 and 14. Therefore, days 7 and 14 were selected to evaluate specific parameters, such as cell viability (live/dead), apoptosis, and oxidative stress—ROS (Figure 1C). Results showed that most cells remained intact (live), in spite of demonstrating signals of apoptosis. Additionally, no ROS was detected. Considering all the parameters tested, the established regimen conditions for subsequent studies were an initial density of 2000 cells/well and incubation for 7 days (Video S1).

#### 3.1.2. BT-20 Cell Line

**Figure 2:**
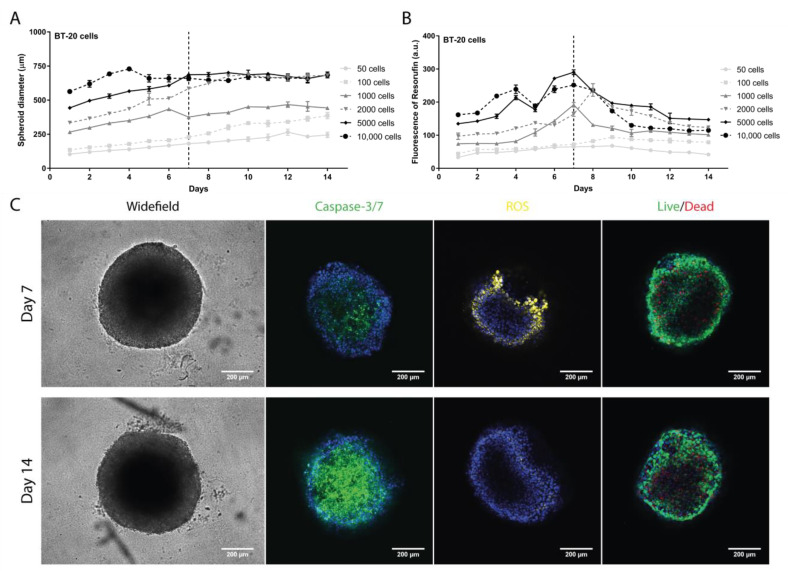
Evaluation of the BT-20 spheroid formation. (**A**) Spheroid diameter (µm) daily monitoring over 14 days measured with a widefield fluorescence microscope. (**B**) Spheroid cell metabolic activity daily evaluation over 14 days by measuring the fluorescence emission of resorufin with a microplate reader. (**C**) Representative images of parameters evaluated on days 7 and 14 at 5000 cells/well with a confocal point-scanning Zeiss LSM 880 microscope. In the apoptosis determination, apoptotic cells are marked in green; in the oxidative stress measurement, reactive oxygen species (ROS) are marked in yellow; and in the cell viability/mortality evaluation, intact cells (live) are marked in green and permeabilized cells (dead) in red. In all experiments, cell nucleus is marked in blue. Scale bar = 200 µm. Graphs represent at least three biological repeats.

For BT-20, the diameter increased over time until day 14 for all initial cell densities with the exception of 10,000 cells/well (Figure 2A). The metabolic activity showed a maximum between days 7 and 8 in the range of 1000 to 10,000 cells/well (Figure 2B). Then, a significant decrease until day 14 was observed. There was a slight increase in the fluorescence intensity up to day 9, followed by a weak decrease for 50 and 100 cells/well. 

The best results were observed with 5000 cells/well, where a gradual increase in the diameter and metabolic activity were observed until day 7, followed by diameter stabilization and decrease in metabolic activity between days 7 and 14. Again, days 7 and 14 were selected to evaluate specific parameters (Figure 2C). Results revealed that most cells were intact, with an increase in production of ROS on day 7, while on day 14 an increase in apoptosis was detected. Based on these results, the regimen conditions for subsequent studies were an initial cell density of 5000 cells/well and a growth period of 7 days (Video S2).

#### 3.1.3. BT-474 Cell Line

**Figure 3:**
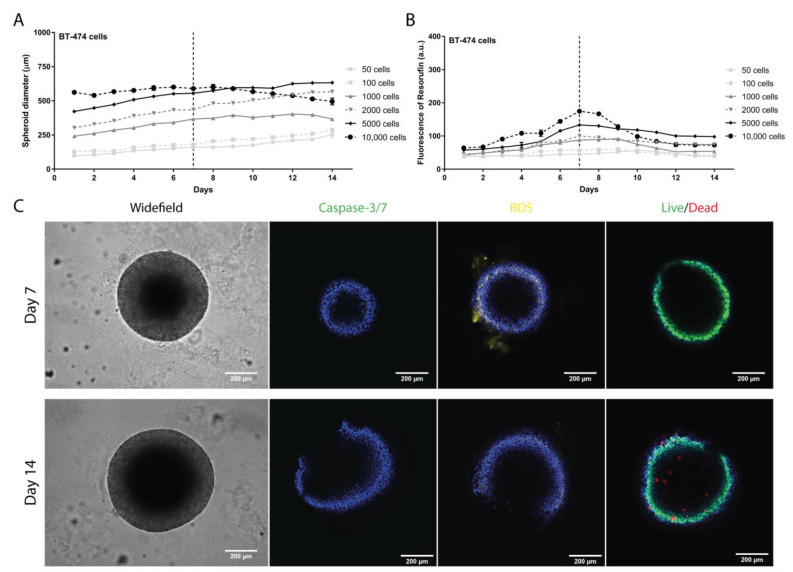
Evaluation of the BT-474 spheroid formation. (**A**) Spheroid diameter (µm) daily monitoring over 14 days measured with a widefield fluorescence microscope. (**B**) Spheroid cell metabolic activity daily evaluation over 14 days by measuring the fluorescence emission of resorufin with a microplate reader. (**C**) Representative images of parameters evaluated on days 7 and 14 at 5000 cells/well with a confocal point-scanning Zeiss LSM 880 microscope. In the apoptosis determination, apoptotic cells are marked in green; in the oxidative stress measurement, reactive oxygen species (ROS) are marked in yellow; and in the cell viability/mortality evaluation, intact cells (live) are marked in green and permeabilized cells (dead) in red. In all experiments, cell nucleus is marked in blue. Scale bar = 200 µm. Graphs represent at least three biological repeats.

BT-474 spheroid diameters showed a steady increase until day 14, except for 10,000 cells/well, with diameters decreasing after day 7 (Figure 3A). As for metabolic activity, there was a maximum on days 7 to 9, followed by a decrease until day 14 (Figure 3B). Again, 5000 cells/well was the initial cell density chosen to proceed, considering the higher diameter and metabolic activity. At selected time-points, days 7 and 14, the parameters of viability, ROS, and apoptosis revealed permeabilization of cells (labeled in red). ROS production was higher on day 7 than on day 14, and apoptosis was negligible (Figure 3C). Based on these results, we considered an initial cell density of 5000 cells/well and a growth period of 7 days as the best regimen conditions (Video S3). 

### 3.2. Ultrastructure of Spheroids

**Figure 4:**
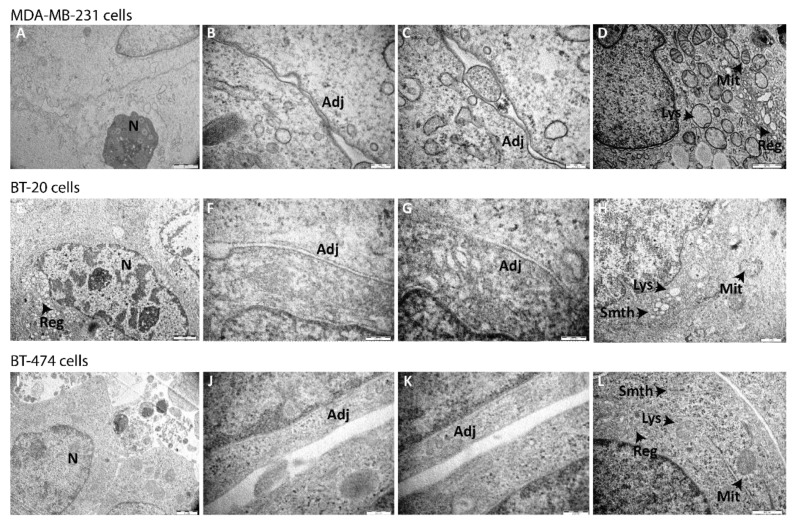
Ultrastructure of MDA-MB-231, BT-20, and BT-474 spheroids by transmission electron microscopy (TEM). Images of (**A**–**D**) MDA-MB-231, (**E**–**H**) BT-20, and (**I**–**L**) BT-474 spheroids formed under optimized conditions were acquired after 7 days of culture with TEM microscope Hitachi H-7000. Scale bars are shown on all images. N = nucleus; Adj = adjoined connections; Mit = mitochondria; Lys = lysosome; Reg = rough endoplasmic reticulum; Smth = smooth endoplasmic reticulum.

The organization and ultrastructure of spheroids were analyzed by TEM in MDA-MB-231 (Figure 4A–D), BT-20 (Figure 4E–H), and BT-474 (Figure 4I–L) under the previously optimized conditions. Low magnification TEM showed cell–cell proximity with sporadic villosities in tissue-like fashion, revealing intact adjoined cells within the spheroid. At higher magnifications, the presence of normal and abnormal phenotype-associated mitochondria was observed, as well as rough and smooth endoplasmic reticulum without abnormal features, and lysosomes. Other organelles, as well as the nuclear membrane, showed typical morphology for the phenotype. 

### 3.3. Peptide Anticancer Activity in Cell Monolayers and Spheroids

The activity of vCPP2319 was evaluated in monolayers of MDA-MB-231, BT-20, and BT-474 cells for 24 h, with the IC_50_ values reported in Table 2. In this case, vCPP2319 demonstrated a high elimination efficiency towards TNBC cell lines, with IC_50_ values around 4.5 µM. The elimination capacity towards BT-474 cells was less marked, with an IC_50_ three times higher. As a control, cytotoxicity was evaluated against PepH3, a well-known blood-brain barrier peptide shuttle (BBBpS) [48,49]. As expected, PepH3 showed no activity up to 100.0 µM, despite its ability to penetrate cell barriers. 

The efficacy of vCPP2319 was also evaluated on spheroids using the previously optimized regimens. After development, spheroids were exposed to increasing peptide concentrations (0.05–100.0 µM) up to 5 days (Figure S1 and Table S1). A stable IC_50_ value was observed upon 5 day incubation (best anticancer activity reported), with results shown in Table 2. Overall, the IC_50_ values of vCPP2319 towards spheroids were higher than for cell monolayers. Again, as in cell monolayers, PepH3 did not show anticancer activity. 

### 3.4. Effect of Peptide Treatment on Spheroids

**Figure 5:**
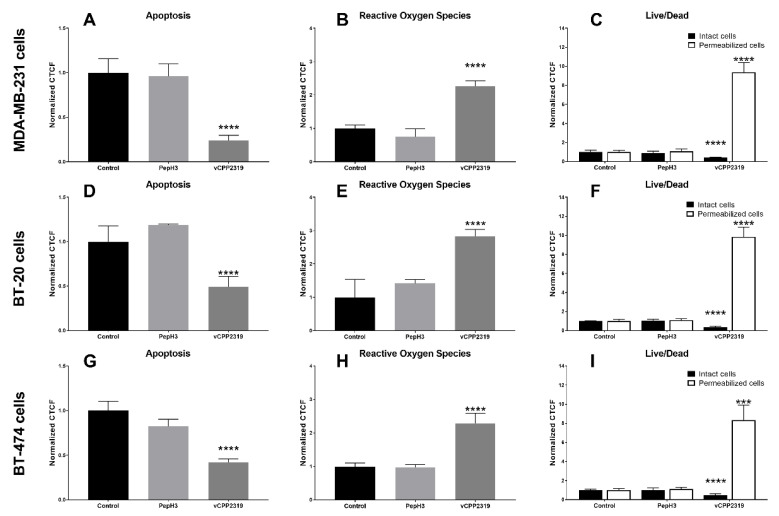
Confocal microscopy characterization of apoptosis, reactive oxygen species (ROS) production, and cell viability/mortality in spheroids after treatment with peptides. Apoptosis, ROS production, and cell viability/mortality of (**A**–**C**) MDA-MB-231, (**D**–**F**) BT-20, and (**G**–**I**) BT-474 spheroids, formed under optimized conditions, after treatment for 5 days with vCPP2319 and PepH3 were assessed using a confocal point-scanning Zeiss LSM 880 microscope. The analysis was performed by calculating the corrected total cell fluorescence (CTCF) of different images and CTCF normalized to control. Graphs represent at least triplicate biological repeats, and results are displayed as mean ± SD, where * *p* < 0.05, ** *p* < 0.01, *** *p* < 0.001, and **** *p* < 0.0001.

The effect of vCPP2319 on apoptosis, ROS production, and cell permeabilization of spheroids was assessed over 5-day incubation at IC_50_ value, followed by quantitative analysis by confocal microscopy (Figure 5). For all spheroids, vCPP2319 treatment caused alterations in cell apoptosis, ROS production, and cell viability. Thus, the peptide significantly decreased apoptotic cells compared to control (*p* < 0.0001 for all cell lines). Then, it significantly increased the fluorescence emission resulting from ROS production compared to the control (*p* < 0.0001 for all cell lines). Finally, vCPP2319 was capable of efficiently permeabilizing cancer cells (dead), as revealed by live/dead assay compared to control (*p* < 0.0001 for all cell lines). PepH3, also used as a control in each assay, did not produce statistically significant alteration in the spheroid microenvironment, despite its ability to penetrate cell barriers. 

## 4. Discussion

The use of 3D cell cultures has attracted increasing interest as being more representative of in vivo conditions than 2D cultures [50]. For cancer research, it is particularly important to consider the unique properties of the tumor microenvironment, which can affect therapeutic efficacy [51,52]. Spheroids can better reproduce tumor hypoxia, expression of extracellular matrix proteins, intracellular interactions, and growth factor exchanges [15,16,17]. In the literature, numerous examples of tumor cell line-derived spheroids can be found using different cancer cell lines, but only some include their evaluation against an anticancer drug [25,28,31,32,33,34,35,36,37,38,39,40,41,53], which is usually a chemotherapeutic agent and not an anticancer peptide, as evaluated in this work. Additionally, a full characterization of the effect of therapeutics on apoptosis and ROS production is sometimes missing.

To develop spheroids representing hard-to-treat BC subtypes, namely TNBC and HER2+, we optimized preparation conditions using three cell lines (MDA-MB-231, BT-20, and BT-474 cells) selected for their importance in in vitro cancer research. The liquid overlay technique, which relies mainly on gravity, was used to generate these spheroids [54]. As extracellular matrix is needed to develop compact and homogeneous spheroids [30,55], 2% (*v*/*v*) GelTrex^®^ LDEV-Free was used [28]. Our results showed that spheroids produced with either 2000 cells/well (MDA-MB-231) or 5000 cells/well (BT-20 and BT-474) reached a stable diameter (below 1 mm) and higher metabolic activity at 7 days (Figure 1). MDA-MB-231 cells displayed a faster expansion than BT-20 and BT-474, in tune with their high proliferative nature [56], thus requiring fewer initial cells. Eventually, the 3D structure reached a senescent state due to lack of nutrients or hypoxia, as shown by the absence of cell death increase in the live/dead assay [57,58]. This was confirmed by evaluation of apoptosis, ROS production, and cell permeabilization (live/dead) (Figure 1, Figure 2 and Figure 3 and Videos S1 and S2). These results are consistent with previous reports [28,39,40]. 

Cellular organization and ultrastructure of spheroids at day 7 were analyzed by TEM. The results showed the presence of intact cells adjoined in a tissue-like fashion. Inside cells, organelles such as mitochondria, rough and smooth endoplasmic reticulum, and lysosomes showed the typical phenotype morphology (Figure 4). 

To assess the cytotoxic activity of vCPP2319, an ACP discovered by our group [43,44], spheroids presenting higher diameter and metabolic activity (day 7) were used. For comparison, vCPP2319 cytotoxicity towards cell monolayers was also tested. Results showed higher antitumor potency in TNBC than in HER2+ cells, with a 2-fold higher IC_50_ value for HER2+ spheroids. IC_50_ value also differed between monolayers and spheroids, in both TNBC (IC_50_ (monolayer) ≈ 4.5 µM after 24 h, vs. IC_50_ (spheroid) ≈ 20.0 µM after 5 days) and HER2+ (IC_50_ (monolayer) ≈ 15.7 µM after 24 h, vs. IC_50_ (spheroid) ≈ 47.9 µM after 5 days) (Table 2). Anticancer activity of vCPP2319 in spheroids was monitored over 5 days and a time-dependent action was observed, with IC_50_ values ranging between 62.0–87.0 µM at 24 h and 21.0–47.0 µM at day 5 (Table S1 and Figure S1). To validate the cytotoxic effect of vCPP2319, PepH3, a BBBpS, was used as negative control due to the lack of anticancer activity [43,48,49]. Results revealed no cytotoxic effect of PepH3 (Table 2 and Figure S1). The differences observed between both models might be related to increased complexity in the spheroid, including cellular heterogeneity and/or resistance phenotypes. Moreover, the ultrastructure, which includes extracellular matrix, likely decreases the peptide’s ability to penetrate cells, reducing its efficacy, similarly to what is observed in tumor response in vivo [59]. 

The cytotoxic effect of vCPP2319 was further demonstrated through evaluation of apoptotic cells, ROS production, and cell permeabilization in control spheroids and spheroids treated with vCPP2319 and PepH3 (Figure 5). Our results revealed increased ROS production and cell permeabilization. Fewer apoptotic cells were visualized in spheroids treated with vCPP2319, when compared with controls, which means that cells were already compromised or were already dead at the time of detection (Figure 5). A decrease in potency has been reported already in other studies [60,61,62]. Based on these findings, we suggest that anticancer drug candidates should be tested in spheroids to properly characterize their efficacy. In addition to those in the present work, other cancer cell lines can be used to better mimic other subtypes.

Taken together, our results have demonstrated the relevance of 3D cell culture models for better representing in vivo tumors. Nevertheless, our study has some differences compared to other studies, such as the lack of co-culture with stromal cells (such as fibroblasts), and the comparison of the efficacy of an anticancer peptide towards spheroids and cell monolayers instead of a well-established chemotherapeutic agent. However, using our optimized spheroids, we showed that the sensitivity of 2D and 3D models to different drugs, such as the ACP, was different; thus, the use of 2D models might not be accurate when considering what happens in the in vivo setting. This highlights the advantage of BC spheroid models as drug screening platforms to afford better chances of success. Considering the recent efforts to develop anticancer peptides, which present advantages over traditional chemotherapy, it is important to show the need for complex models to properly assess peptide activity. 

## 5. Conclusions

Three-dimensional cell culture models are increasingly viewed as essential for the preclinical evaluation of drug candidates (particularly anticancer) as they are closer to the in vivo setting than 2D cultures. During R&D on new therapeutic molecules, testing their efficacy in 3D models avoids overestimation of cytotoxic efficacy. In this study, we successfully determined the best regimen for developing TNBC spheroids from MDA-MB-31 and BT-20 cell lines, and HER2+ spheroids from BT-474. The characterization of each spheroid revealed different tumor microenvironment properties. We also compared cell monolayers and spheroids in their response to an ACP. The spheroids were less sensitive than the monolayers to the peptide tested, which illustrates the need for preclinical models that properly mimic the in vivo setting. The spheroids in this study are valuable tools for preclinical assessment of newly developed anticancer drugs. 

## Figures and Tables

**Table 1:** Peptides used in this study.

Peptide	Amino Acid Sequence	Mass (Da), Calculated (Found)	HPLC t_R_ (min)	Purity (%) ^1^
PepH3	AGILKRW-amide	842.8 (843.0)	5.5	99.5
vCPP2319	WRRRYRRWRRRRRWRRRPRR-amide	3179.8 (3180.2)	6.9	99.1

^1^ Peptide purity was estimated by peak integration of the analytical HPLC chromatograms. Da, dalton; t_R_, retention time.

**Table 2:** Anticancer activity of peptides in cell monolayers and spheroids.

Peptide	IC_50_ Values [μM]					
	TNBC Cells				HER2+ Cells	
	MDA-MB-231		BT-20		BT-474	
	Monolayer *	Spheroid ^#^	Monolayer *	Spheroid ^#^	Monolayer *	Spheroid ^#^
PepH3	>100	>100	>100	>100	>100	>100
vCPP2319	4.5 ± 0.07	22.1 ± 3.67	4.2 ± 2.22	21.3 ± 2.98	15.7 ± 1.46	47.9 ± 3.97

IC_50_ is the concentration causing 50% death of cells. HER2, Human epidermal growth factor receptor-2; TNBC, Triple-negative breast cancer; *, results upon 24-h incubation; ^#^, results upon 5-day incubation.

## Data Availability

Not applicable.

## Supplementary Materials

The following are available online at https://www.mdpi.com/article/10.3390/pharmaceutics13111863/s1, Table S1: Anticancer activity of peptides on 3D cell cultures over 5 days, Figure S1: In vitro cytotoxicity of peptides towards different cancer cell lines with an incubation up to 5 days, Video S1: Development of MDA-MB-231 cell line spheroids, Video S2: Development of BT-20 cell line spheroids, Video S3: Development of BT-474 cell line spheroids.
