# Supplementary material for: Development of Breast Cancer Spheroids to Evaluate Cytotoxic Response to an Anticancer Peptide
Source: Pharmaceutics. 2021 Nov 4;13(11):1863. doi: 10.3390/pharmaceutics13111863 (PMC8619419; doi:10.3390/pharmaceutics13111863)
Supplement: Supplementary file 1 [file pharmaceutics-13-01863-s001.zip › pharmaceutics-1431613-supplementary.pdf]

pharmaceutics

MDPI

Supplementary Materials: Development of Breast Cancer Spheroids to Evaluate Cytotoxic Response to an Anticancer Peptide

Marco Cavaco, Patrícia Fraga, Javier Valle, David Andreu, Miguel A.R.B. Castanho and Vera Neves

Table S1. Anticancer activity of peptides on 3D cell cultures over 5 days.

Peptide	Cancer Cell Lines	IC_{50} [μM]				
		Day 1	Day 2	Day 3	Day 4	Day 5
PepH3	MDA-MB-231	> 100	> 100	> 100	> 100	> 100
	BT-20	> 100	> 100	> 100	> 100	> 100
	BT-474	> 100	> 100	> 100	> 100	> 100
vCPP2319	MDA-MB-231	69.8 ± 3.34	42.8 ± 1.45	34.7 ± 1.51	25.2 ± 0.94	22.1 ± 3.67
	BT-20	62.2 ± 3.76	46.5 ± 3.36	34.9 ± 2.32	28.6 ± 1.23	21.3 ± 1.98
	BT-474	87.2 ± 5.37	77.9 ± 2.76	61.3 ± 2.28	51.9 ± 1.72	47.9 ± 3.97

IC_{50} is the concentration causing 50% death of cells.

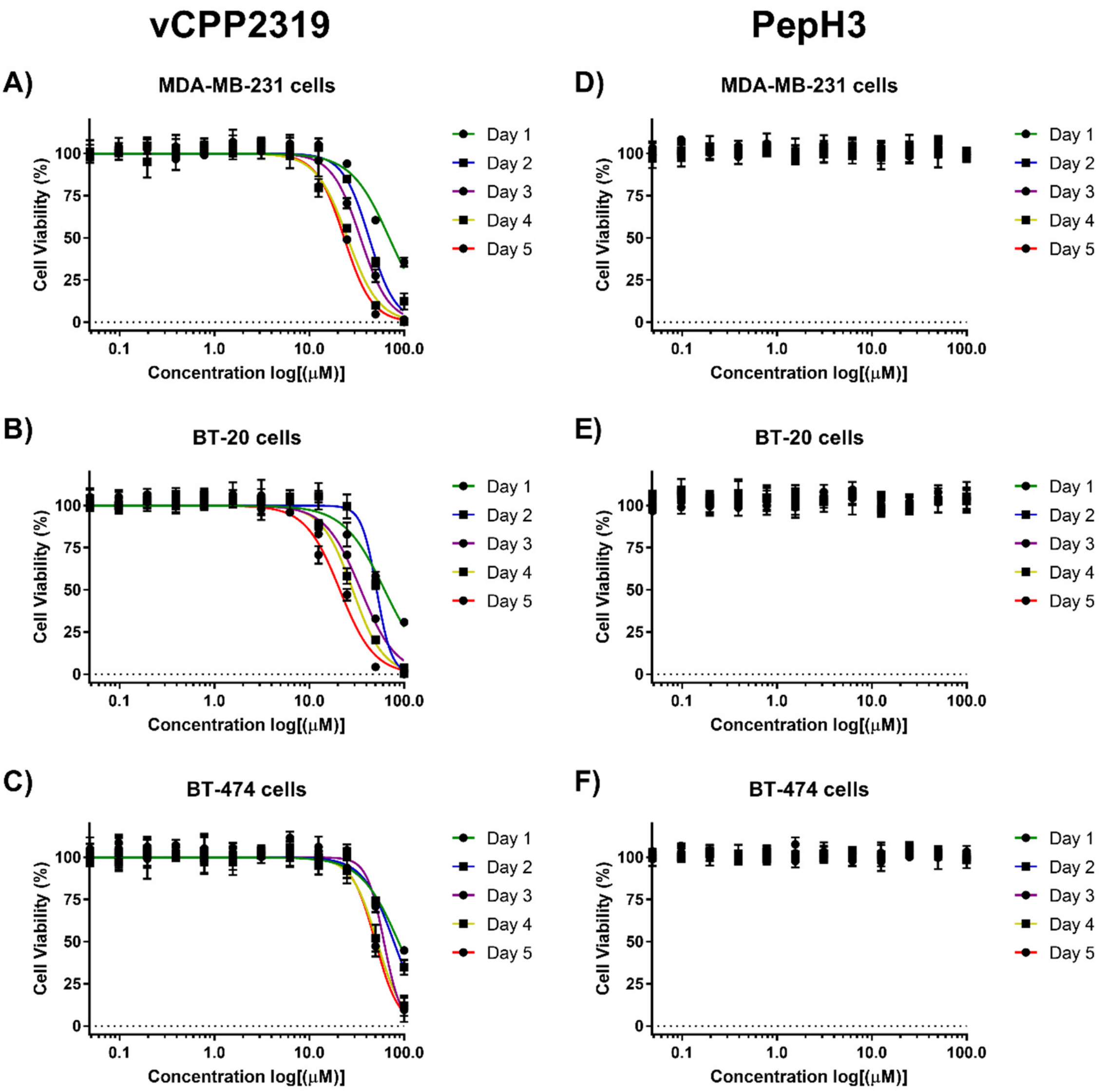

Figure S1. In vitro cytotoxicity of peptides towards different cancer cell lines with an incubation up to 5 days**.** For MDA-MB-231 cells (**A** and **D**), an initial cell density of 2 000 cells/well and an incubation of 7 days was used; and for BT-20 (**B** and **E**) and BT-474 cells (**C** and **F**), an initial cell density of 5 000 cells/well and an incubation of 7 days was used. After spheroid formation, cells were treated with increasing concentrations of vCPP2319 (**A**–**C**) and PepH3 (**D**–**F**) (0.05–100.0 μM range, in medium) up to 5 days in culturing conditions without medium change, and cell viability was assessed using CellTiter-Blue® Cell Viability Assay. IC_{50} values were determined using the GraphPad Prim 7.0 software (GraphPad Software, San Diego, CA, USA) using a log(inhibitor) vs. normalized response. Experiments were performed at least three times on different days using independently grown cell cultures.
